# Development and validation of a questionnaire about fluoride knowledge

**DOI:** 10.1590/0103-644020256377

**Published:** 2025-08-11

**Authors:** Ana Beatriz Chevitarese, Karla Lorene de França Leite, Kenderson Santos Silva, Lucas Alves Jural, Deborah Rachel Caldas da Rocha, Matheus França Perazzo, Jaime Aparecido Cury, Lucianne Cople Maia

**Affiliations:** 1 School of Dentistry, Department of Pediatric Dentistry and Orthodontics, Universidade Federal do Rio de Janeiro (UFRJ), Rio de Janeiro, RJ, Brazil.; 2 School of Dentistry, Department of Oral Health, Universidade Federal de Goiás (UFG), Goiania, GO, Brazil.; 3 Piracicaba Dental School, Universidade Estadual de Campinas (UNICAMP), Piracicaba, SP, Brazil

**Keywords:** Fluorides, Surveys and questionnaires, Dentists, Health personnel, Psychometry

## Abstract

Objective: To develop and validate a questionnaire about fluoride knowledge (FKQ). Methods: Questions to evaluate knowledge about fluoride sources and daily fluoridated water and dentifrices consumption were developed and validated. Sociodemographic data were also collected from the participants, and data were collected for testing their psychometric properties and instrument validation. The model adjustment was tested in 456 dentists, students, and health personnel via confirmatory factor analysis (CFA) with unidimensional categorical indicators. Cronbach’s alpha coefficient was used to estimate the instrument’s reliability. External validation measurements were represented by questions related to sociodemographic data, fluoride source knowledge and daily practices, and the consumption of fluoride water and dentifrices. Results: The model showed adequate fit via confirmatory factor analysis (CFA); the fit indices were comparative fit index (CFI) = 0.941, Tucker‒Lewis index (TLI) = 0.927, and root mean square error of approximation (RMSEA) = 0.075, with factorial loadings varying between 0.25 and 0.91. The Cronbach's alpha coefficient for the 13 items was 0.68, demonstrating substantial and adequate reliability in the total instrument. Conclusion: The FKQ can estimate the knowledge of dentists, dental students, and health personnel about fluoride use.

## Resumo

Objetivo: Desenvolver e validar um questionário sobre conhecimento em fluoreto (FKQ).Métodos: Foram elaboradas e validadas questões para avaliar o conhecimento sobre fontes de fluoreto e o consumo diário de água fluoretada e dentifrícios. Também foram coletados dados sociodemográficos dos participantes, além de informações para testar as propriedades psicométricas e a validação do instrumento. O ajuste do modelo foi testado em 456 dentistas, estudantes e profissionais de saúde por meio da análise fatorial confirmatória (CFA) com indicadores categóricos unidimensionais. O coeficiente alfa de Cronbach foi utilizado para estimar a confiabilidade do instrumento. As medidas de validação externa foram representadas por questões relacionadas aos dados sociodemográficos, conhecimento das fontes de fluoreto, práticas diárias e consumo de água fluoretada e dentifrícios. Resultados: O modelo apresentou um ajuste adequado na análise fatorial confirmatória (CFA), com índices de ajuste: índice de ajuste comparativo (CFI) = 0,941, índice de Tucker-Lewis (TLI) = 0,927 e erro médio quadrático de aproximação (RMSEA) = 0,075, com cargas fatoriais variando entre 0,25 e 0,91. O coeficiente alfa de Cronbach para os 13 itens foi de 0,68, demonstrando confiabilidade substancial e adequada para o instrumento como um todo.Conclusão: O FKQ pode estimar o conhecimento de dentistas, estudantes de odontologia e profissionais de saúde sobre o uso do fluoreto.

**Figure:**
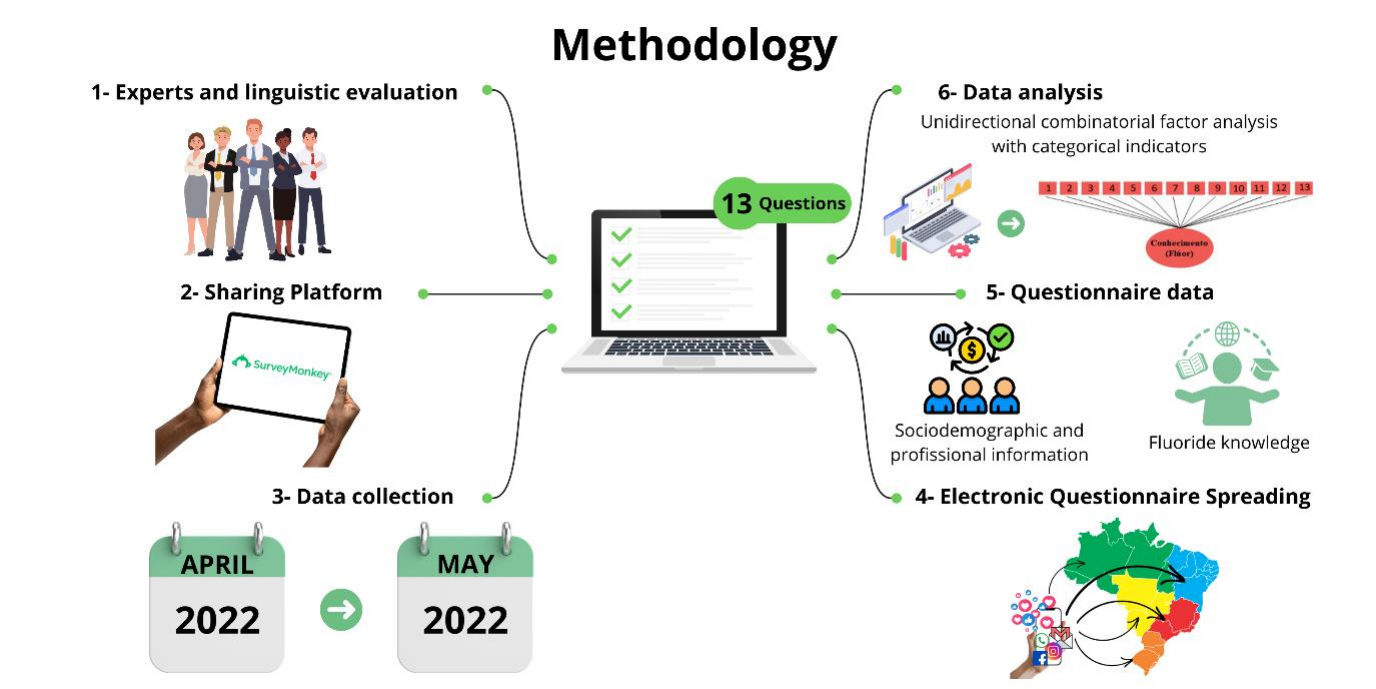

## Introduction

Dental caries is still a significant public health challenge, impacting millions worldwide, especially in socially disadvantaged families ^1^ ^,^ ^2^. Although sugar is the pivotal cause of caries, control of this disease has focused on improving oral hygiene habits and fluoride use ^3^.

Although the efficacy and safety of fluoride use for caries control is based on scientific evidence, the spread of false or misleading content about fluoride sometimes discourages its use based on possible adverse health effects ^4^. This scenario supports the development of dental beliefs that could negatively impact oral health behaviors ^5^. Moreover, fluoride refusal is a growing concern in dental offices and is likely driven or reinforced by internet-based disinformation ^6^. Indeed, diverse aspects such as innovative messages, information overload, and predisposed personal characteristics such as preexisting beliefs, ideological motivations, and political polarization influence the spread of false or misleading web-based content ^7^ ^,^ ^8^ ^,^ ^9^ ^,^ ^10^.

Therefore, the use of an instrument to estimate dentists’, students’, and health personnel's knowledge of fluoride sources and daily consumption of fluoride water and dentifrices with a validated instrument is urgently needed. Health-metering instruments play an important role in public health, clinical practice, and decision-making processes for health service organizations ^11^. In dentistry, several instruments have been developed and used to assess the impact that oral diseases or even poor oral health have on daily functioning, and these data are a useful source of information for planning and organizing oral health care services directed to the studied population ^12^.

Therefore, the development of a robust instrument that can measure this knowledge is necessary to evaluate how prepared these dental providers are to instruct their patients correctly about fluoride sources, which is why this study aimed to develop a questionnaire about fluoride knowledge (FKQ) and assess its psychometric properties among Brazilian dentists, students, and health personnel. 

## Materials and methods

The study was approved by the Research Ethics Committee of Clementino Fraga Filho University Hospital (Federal University of Rio de Janeiro). All participants signed informed consent forms to take part in the study.

### Study design and participants

The study design and protocol were based on those of Jural et al. ^2021^ ^) (^ ^13^. This Brazilian cross-sectional study included dentists, students, and oral health personnel (dental hygienists and dental assistants) with professional registration at their respective regional dentistry councils, comprising age groups of both genders from all regions of the country, as eligibility criteria. The participants answered an electronic questionnaire hosted by SurveyMonkey Inc. (San Mateo, USA) and spread by WhatsApp, Instagram, Facebook, Linked In, Twitter, and Telegram, which tested professionals’ knowledge about measures of fluoride sources and daily practices and consumption of fluoride water and dentifrices. The snowball sampling data were collected from April-May 2022 to test the psychometric properties of the FKQ.

The questionnaire was divided into two parts: 8 questions on sociodemographic and professional information and 13 questions on measures of fluoride sources and daily practices and the consumption of fluoride water and dentifrices. The available scientific evidence was consulted to establish the correct answer for each question in the instrument.

### Questionnaire development and assessment of psychometric properties

A questionnaire was developed, and its properties were evaluated through the following steps: a) elaboration of the questions; b) expert’s technical evaluation; c) general evaluation by an expert committee; d) linguistic evaluation; and e) psychometric evaluation.

### Elaboration of the questions

The instrument was divided into two parts: a first questionnaire about sociodemographic questions (age, gender, scholarly level, and state of residence) and a second questionnaire with questions to measure knowledge about fluoride sources and the daily consumption of fluoride water and dentifrices.

Initially, a data search was performed at PubMed, Scopus, and Embase to identify systematic reviews and randomized controlled studies for interventional studies, observational studies, systematic reviews, cohort studies, case-control studies, and transverse studies were selected. After this search, two dentistry students, three pediatric dentists, and one orthodontist developed and designed questions about measures of fluoride sources and daily practices and the consumption of fluoride water and dentifrices by dentistry students, dentists, and oral health personnel, with a binary response format (YES or NO).

### Expert’s technical evaluation

The questions were submitted to a dentist and chemical expert in the fluoride field. This professional was asked if the developed questions would effectively assess the students’, dentists’, and oral health personnel's knowledge about measures of fluoride sources and daily practices and consumption of fluoride water and dentifrices. The suggestions were incorporated into the instrument.

### Evaluation by the expert committee

The questionnaire was evaluated by a committee of six specialists, PhDs in dentistry, with recognized expertise in their respective areas, and the committee judged each item individually and classified it as “inadequate”, “partially adequate”, or “inadequate”. The developers observed the judges’ suggestions and modified them until a consensus was reached. At this point, in addition to the dental technical aspects, the clarity and comprehensibility of the questionnaire were observed.

### Linguistic evaluation

The involvement of a journalist in the linguistic assessment of the instrument was a deliberate choice aimed at ensuring clarity, coherence, and accessibility, as well as enhancing the linguistic and grammatical aspects of the questionnaire.

### Assessment of psychometric evidence

An instrument containing sociodemographic and professional information and information about measures of fluoride sources, daily practices, fluoride water consumption, and dentifrices outcomes was self-administered. The sample consisted of 456 participants, a size determined to ensure a robust psychometric evaluation through confirmatory factor analysis (CFA). Unlike traditional hypothesis-driven studies, psychometric research requires a sample size that accounts for model complexity, the number of estimated parameters, and the distribution of factor loadings rather than relying on arbitrary participant‒to-item ratios. Prior to data collection, a second pilot study was conducted with 15 participants to assess the methodological approach and participant compliance. Based on these findings, minor adjustments were made, and these pilot participants were excluded from the final analysis.

### Statistical analysis data analysis plan

Data handling and statistical analysis were performed via SPSS v. 23.0 and Mplus v. 8.8. Sample characterization was performed according to professional category, gender, and age. The descriptive analysis was applied to score the right answers in each Brazilian country region, and the chi-square (χ²) test was used to determine the construct representativity.

The internal structure of the questionnaire was assessed for model fit by the chi-square (χ²) test, the comparative fit index (CFI), the Tucker‒Lewis index (TLI), and the root mean squared error of approximation (RMSEA) [14]. The following thresholds were adopted to adjust the model fit: χ² < 0.05; CFI > 0.90; TLI > 0.90; and RMSEA < 0.06 for acceptable fit [15].

The reliability of the instrument’s final version was determined on the basis of internal consistency, which was estimated via Cronbach’s alpha coefficient. Values ≥ 0.7 were considered substantial and adequate ^14^.

## Results

The adjustment model was tested via unidimensional confirmatory factor analysis (CFA) with categorical indicators and model fit indices: CFI = 0.941, TLI = 0.927, RMSEA = 0.075 (0.064-0.086) and factor loadings varying from 0.25--0.91. External measures of validation were represented by related questions of sociodemographic data, knowledge about fluoride sources and daily practices and consumption of fluoride water and dentifrices. Thirteen questions were included in the analysis, and the results were adequate or acceptable, as shown in Table 1.

**Table 1:** Questions from the fluoride construct and their respective factor loadings

Questions	Factor loadings (CI 95%)
1. Is fluoride naturally present in water?	0.46 (0.34-0.58)
2. Is fluoride naturally present in food?	0.63 (0.52-0.75)
3. In Brazil, are the states that have water treatment stations obligated to adjust the fluoride content in the water supply?	0.43 (0.28-0.58)
4. Drinking fluoridated water may cause cancer?	0.85 (0.74-0.96)
5. Does drinking fluoridated water reduce dental caries evelopment?	0.71 (0.59-0.84)
6. Do black tea leaves have natural fluoride content?	0.66 (0.54-0.84)
7. Do green tea leaves have natural fluoride content?	0.72 (0.60-0.85)
8. Does fluoride modify water’s taste?	0.86 (0.80-0.94)
9. Does fluoride modify water’s color?	0.91 (0.83-0.99)
10. Does fluoridated water affect human intelligence?	0.66 (0.50-0.83)
11. Should we brush our teeth with fluoridated dentifrice to improve dental caries resistance?	0.53 (0.34-0.72)
12. Could children and their parents use the same fluoridated dentifrice?	0.44 (0.32-0.56)
13. Could the amount of fluoridated dentifrice used by children to brush the teeth is made in accordance with their age?	0.25 (0.01-0.49)

In the analysis of internal consistency, the Cronbach’s alpha coefficient for the 13 self-reported items was 0.68, which can be considered indicative of reliability since the value is very close to 0.7.

Four hundred forty-nine participants answered the questionnaire in full, where 342 (76.3%) were dentists (mean age =44 y/o; min=23 and max=74), 85 (18.9%) were students (mean age =22 y/o; min=18 and max=48), and 22 (4.8%) were health personnel (mean age =30.5 y/o; min=17 and max=62). Most participants were female (80.7%). The participants were split into five Brazilian regions, as shown in Table 2, in which the weighted average of correct answers was taken and a score was produced for each country region.

Thirteen questions are listed in Table 3, and the respondents were divided according to their gender and professional category. For each question, there must be two options for the answer (YES/NO), and the correct answers or mistakes of each one were considered right/wrong. A significant difference existed between the groups only for the variable’s students and for questions #3, #4, #5, #8, #9, #10, #11 and #13.

Regarding professional category and age (Table 4), we found significant differences for the dentist (#1, #6 and #7), student (#5 and #12) and health personnel groups (#2, #3, #4, #8, #9, #10 and #12).

**Table 2:** Survey sample according to professional category, Brazilian country region and score of correct answers.

Regions	Dentists (n=342)		Students (n=85)		Health Personnel (n=22)	
	n (%)	Score	n (%)	Score	n (%)	Score
Northeast	70 (20.4%)	9.4 ± 2.1	24 (32%)	8.7 ± 1.4	01 (4.5%)	8.0 ± 0.0
North	18 (5.2%)	9.5 ± 1.6	02 (2.3%)	6.0 ± 0.0	01 (4.5%)	5.0 ± 0.0
South	41 (11.9%)	10.4 ± 1.8	09 (10.5%)	8.0 ± 1.5	02 (9.0%)	8.0 ± 0.0
Southeast	160 (46.7%)	10.0 ± 2.1	25 (29.4%)	8.6 ± 1.8	17 (77.2%)	6.8 ± 3.6
Mid-west	53 (15.4%)	9.8 ± 1.8	25 (29.4%)	8.6 ± 1.9	01 (4.5%)	8.0 ± 0.0

Score= number of correct answers for each group

**Table 3:** Survey sample according to professional category and gender.

.**Questions**	Gender	Dentist			Students			Health Personnel		
		Right	Wrong	p value	Right	Wrong	p value	Right	Wrong	p value
1	M	30 (44.2%)	38 (55.8%)	0.501	5 (29.4%)	12 (70.5%)	0.637	0	01 (100%)	0.262
	F	136 (48.8%)	142 (51.2%)		14 (20.5%)	54 (79.4%)		12 (57.1%)	9 (42.9%)	
2	M	39 (57.3%)	29 (42.6%)	0.542	4 (23.5%)	13 (76.4%)	0.510	0	01 (100%)	0.629
	F	174 (62.7%)	104 (37.2%)		24 (35.2%)	44 (64.7%)		4 (19%)	17 (81%)	
3	M	56 (85.2%)	12 (17.6%)	0.770	17 (100%)	0	0.003*	1 (100%)	0 (0%)	0.484
	F	237 (85.3%)	41 (14.6%)		61 (89.7%)	7 (10.2%)		14 (66.7%)	7 (33.3%)	
4	M	68 (100%)	00 (0%)	0.243	15 (88.2%)	2 (11.8%)	0.001*	1 (100%)	0 (0%)	0.439
	F	267 (96%)	11 (4.0%)		65 (95.6%)	3 (4.4%)		13 (61.9%)	8 (38.1%)	
5	M	64 (94.1%)	4 (5.8%)	0.925	14 (88.4%)	3 (17.6%)	0.023*	1 (100%)	0 (0%)	0.439
	F	259 (93.1%)	19 (6.8%)		61 (89.7%)	7 (10.3%)		13 (61.9%)	8 (38.1%)	
6	M	21 (30.8%)	47 (69.1%)	0.515	3 (17.6%)	14 (82.4%)	0.536	0 (0%)	01 (100%)	0.684
	F	103 (36.9%)	175 (62.7%)		6 (8.8%)	62 (91.2%)		3 (14.3%)	18 (85.7%)	
7	M	17 (25%)	51 (75.5%)	0.338	2 (11.8%)	15 (88.2%)	0.801	0 (0%)	01 (100%)	0.746
	F	93 (33.3%)	185 (66.6%)		5 (7.4%)	63 (92.6%)		02 (9.5%)	19 (90.5%)	
8	M	64 (94.1%)	04 (5.8%)	0.855	13 (76.5%)	4 (23.5%)	0.004*	01 (100%)	0 (0%)	0.484
	F	257 (92.4%)	21 (7.5%)		63 (92.6%)	5 (7.4%)		14 (66.7%)	07 (33.3%)	
9	M	67 (91.5%)	01 (1.4%)	0.257	17 (100%)	0	0.00*	01 (100%)	0 (0%)	0.531
	F	260 (93.5%)	18 (6.4%)		67 (98.5%)	1 (1.5%)		15 (71.4%)	06 (28.6%)	
10	M	65 (95.5%)	03 (4.4%)	0.801	16 (94.1%)	1 (5.9%)	0.011*	01 (100%)	0 (0%)	0.531
	F	270 (97.1%)	08 (2.8%)		61 (89.7%)	7 (10.3%)		15 (71.4%)	06 (28.6%)	
11	M	65 (95.5%)	03 (4.4%)	0.934	17 (100%)	0	0.005*	1 (100%)	0 (0%)	0.629
	F	268 (96.4%)	10 (3.5%)		60 (88.2%)	8 (11.8%)		17 (81%)	04 (19%)	
12	M	44 (64.7%)	24 (35.2%)	0.582	6 (35.3%)	11 (64.7%)	0.607	0	01 (100%)	0.484
	F	166 (59.8%)	112 (40.1%)		29 (42.6%)	39 (57.4%)		7 (33.3%)	14 (66.7%)	
13	M	63 (92.6%)	05 (7.3%)	0.481	15 (88.2%)	2 (11.8%)	0.006*	1 (100%)	0 (0%)	0.629
	F	267 (96%)	11 (4%)	63 (92.6%)	5 (7.4%)	17 (81%)		04 (19%)		

M means male; F means female. * χ*²* Statistical differences (p<0.05).

**Table 4:** Characteristics of survey sample according to professional category and age.

Questions	Age	Dentists			Students			Health Personnel		
		Right	Wrong	p value	Right	Wrong	p value	Right	Wrong	p value
1	≤ med	75 (21.7%)	106 (30.6%)	0.015*	12 (14.1%)	37 (43.5%)	0.581	04 (18.2%)	07 (31.8%)	0.087
	> med	90 (26%)	75 (21.7%)		7 (8.2%)	29 (34.1%)		08 (36.4%)	03 (13.6%)	
2	≤ med	112 (32.4%)	69 (19.9%)	0.899	17 (20%)	32 (37.6%)	0.688	0	11 (50%)	0.027*
	> med	101 (26.2%)	64 (18.5%)		11 (12.9%)	25 (29.4%)		04 (18.2%)	07 (31.8%)	
3	≤ med	155 (48.85%)	26 (7.55%)	0.606	46 (54.1%)	3 (3.5%)	0.408	05 (22.7%)	06 (27.3%)	0.022*
	> med	138 (39.9%)	27 (7.8%)		32 (37.6%)	4 (4.7%)		10 (45.5%)	01 (4.5%)	
4	≤ med	173 (50%)	8 (2.3%)	0.168	46 (54.1%)	3 (3.5%)	0.913	04 (18.2%)	07 (31.8%)	0.008*
	> med	162 (46.8%)	3 (0.9%)		34 (40%)	2 (2.4%)		10 (45.5%)	01 (4.5%)	
5	≤ med	165 (47.7%)	16 (4.6%)	0.086	47 (55.3%)	2 (2.4%)	0.010*	05 (22.7%)	06 (27.3%)	0.076
	> med	158 (45.7%)	07 (2.0%)		28 (32.9%)	8 (9.4%)		09 (40.9%)	02 (9.1%)	
6	≤ med	53 (15.4%)	129 (37.1%)	0.013*	6 (7.1%)	43 (50.6%)	0.562	01 (4.5%)	10 (45.5%)	0.534
	> med	69 (20%)	95 (27.5%)		3 (3.5%)	33 (38.8%)		02 (9.1%)	09 (40.9%)	
7	≤ med	47 (13.6%)	134 (38.7%)	0.020*	5 (5.9%)	44 (51.8%)	0.441	0	11 (50%)	0.138
	> med	62 (17.9%)	103 (29.8%)		2 (2.4%)	34 (40%)		02 (9.1%)	09 (40.9%)	
8	≤ med	166 (48%)	15 (4.3%)	0.424	45 (52.9%)	4 (4.7%)	0.397	05 (22.7%)	06 (27.3%)	0.022*
	> med	155 (44.8%)	10 (2.9%)		31 (36.5%)	5 (5.9%)		10 (45.5%)	01 (4.5%)	
9	≤ med	169 (48.8%)	12 (3.5%)	0.330	48 (56.5%)	1 (1.2%)	0.389	06 (27.3%)	05 (22.7%)	0.056*
	> med	158 (45.7%)	07 (2.0%)		36 (42.4%)	0		10 (45.5%)	01 (4.5%)	
10	≤ med	177 (51.2%)	04 (1.2%)	0.282	44 (51.8%)	5 (5.9%)	0.770	6 (27.3%)	05 (22.7%)	0.056*
	> med	158 (45.7%)	07 (2.0%)		33 (38.8%)	3 (3.5%)		10 (45.5%)	01 (4.5%)	
11	≤ med	172 (49.7%)	09 (2.6%)	0.213	44 (51.8%)	5 (5.9%)	0.770	09 (40.9%)	02 (9.1%)	1
	> med	161 (46.5%)	04 (1.2%)		33 (38.8%)	3 (3.5%)		09 (40.9%)	02 (9.1%)	
12	≤ med	137 (39.5%)	63 (18.2%)	0.117	15 (17.6%)	34 (40%)	0.021*	01 (4.5%)	10 (45.5%)	0.022*
	> med	75 (21.7%)	71 (20.5%)		20 (23.5%)	16 (18.8%)		06 (27.3%)	05 (22.7%)	
13	≤ med	154 (44.5%)	09 (2.6%)	0.747	46 (54.1%)	3 (3.5%)	0.408	10 (45.5%)	01 (4.5%)	0.269
	> med	176 (50.8%)	07 (2%)	32 (37.6%)	4 (4.7%)	08 (36.4%)		03 (13.6%)		

Med= mean age. * χ*²* Statistical differences (p<0.05)

## Discussion

The construct identified in the questionnaire was fluoride knowledge. All 13 items were correlated with their factor and expressed dentists', students', and health personnel's knowledge of fluoride effects, consumption, and utilization in dentistry. Although the sample was restricted to the Brazilian population, the instrument was able to measure the comprehension of these subjects and may be an applicable and useful tool in subsequent studies.

With respect to sample size, statistical power and sample size can be determined after data collection is called post hoc or retrospective ^15^, and the critical N (CN) statistic for the evaluation of sample size, where a CN ≥ 200, is considered adequate ^16^. After data collection and model specification, we can estimate the post hoc sample power of our sample.

CFA is generally a large-sample technique ^17^, but as a rule, models with robust parameter estimates and variables with high reliability may require smaller samples ^18^. Additionally, the issue of whether the sample size is adequate for achieving the desired power for significance tests, overall model fit, and likelihood ratio tests for specific model/research circumstances is a different aspect considered during power analysis ^19^ ^,^ ^20^. 

Temporal stability (test-retest evaluation) could not be applied in this work because the aim of the construct is fluoride knowledge measurement, and there is no consensus in the literature about the test-retest interval for this study profile. Since the instrument developed in this study was an assessment of knowledge rather than of the participant’s current health status, the slightly longer interval does not affect the current data. In this sense, if the questionnaire was retested, participants could have been interested in looking for the right answers after answering the questionnaire a second time ^21^.

The preliminary value of Cronbach’s alpha coefficient was limited, indicating that the instrument tends to have good properties. According to Landis & Koch ^14^, the internal consistency of a construct varying between 0.6 and 0.8 is substantial, with 0.7 being an acceptable value. Hora et al. ^22^ suggested that a questionnaire should be applied to a significant and heterogeneous population sample (if questionnaires are applied only to specialists in a given area, for example, there will be a natural tendency toward low variance, thus lowering the α), which could explain the Cronbach’s alpha coefficient value found in our study. Additionally, question 13 had a very low factor loading, and if we removed it from the instrument, the value of Cronbach’s alpha coefficient would probably have increased. However, we decided to address this question because once we evaluated its content, we believed that its application in different populations was very important.

All the results of the unidimensional confirmatory factor analysis (CFA) with categorical indicators and model fit indices, such as the comparative fit index and Tucker‒Lewis index, presented values close to 1, in addition to the root mean square error of approximation, with an acceptable value near 0.8. These findings indicate that the construct had validity with respect to the previous theoretical structure of the observed variable set ^23^.

An analysis of our samples revealed that the majority of the respondents were female and from Southeast China, which is a Brazilian region with a high concentration of dentistry schools and a large number of dentists in practice ^24^. We have found that most of the respondents ignore the fact that fluoride is naturally present in the water (57%), as black and green teas (70% and 74%, respectively) have high fluoride contents in their leaves, although this knowledge is well supported by the literature ^25^ ^,^ ^26^. This condition might be negative because dentists and undergraduate students should have learned that drinking and cooking water is naturally fluoridated ^26^, as the tea leaves (black and green) that many people use daily in infusions ^20^. This fluoride content may be very useful for preventing dental caries but can be undesirable when its intake may increase the risk of developing dental fluorosis ^27^.

Moreover, questions about fluoride consumption reflected the poor understanding of its natural presence in water, food, or tea and the effects of drinking fluoridated water or the use of fluoridated dentifrice by the respondents. These questions were consistent with the construct used in measurements of dental providers’ knowledge about fluoride and revealed the lack of information about the natural presence of fluoride in water and tea leaves as the correct prescription of fluoridated dentifrice for children. This fluoride consumption misconception was observed in all three groups analyzed (dentists, students, and health personnel), and a greater percentage of wrong answers was observed among the youngest respondents who were under graduation or had just graduated.

When the age factor was analyzed, it was verified among dentists and students that the older the respondent was, the greater their knowledge regarding fluoride sources and daily practices and the greater their consumption of fluoride water and dentifrices. This fact leads us to presume that students might leave dental schools unprepared or that the degree of teaching in dental schools has worsened over the past few years with the increasing number of new dentistry schools throughout the country. On the other hand, it seems that older dentists may improve their educational learning in postgraduate courses and could apply this knowledge in their clinical practice.

Another important circumstance to be discussed is the need to realize that dental training, in many regions, has proven to be insufficient and inadequate to expand oral health to the majority of the population, with little social impact on public and collective programs ^28^. Although clinicians can meet individual and private needs with recognized technical quality, there is a challenge in terms of comprehensiveness: there is a distance between dentistry graduation teaching and the perspective of oral health universalization facing the real demands of Brazil ^28^.

Another controversial question when we analyzed the questionnaire’s answers was *“Should children and their parents use the same fluoridated dentifrice?*”. Practically half of the students and health personnel answered that this affirmative was wrong. This wrested idea corroborates the fallacy supported by the dentifrice industry’s sturdy marketing to sell toothpaste for an infant niche and shows the reality that these future dentists are not receiving good and proper training in dental schools about fluoride sources and their importance in controlling dental caries. This could be a problem depending on the child’s age, whereas the recommendation for caries prevention in toddlers is to promote parent-performed brushing twice daily with fluoridated toothpaste containing 1000-1500 ppm NaF ^29^. Considering the data currently available on the anticaries effect of toothpaste with different fluoride concentrations on children and adolescents or on the primary dentition of preschool children, the only scientific-based recommendation is that a small amount of toothpaste with at least 1000 ppm F be used, irrespective of the child’s age ^29^.

## Conclusion

In summary, the validated questionnaire about fluoride knowledge (FKQ) was psychometrically appropriate for use in the Brazilian context, and the evidence of its internal structure confirmed its theoretical basis for measuring fluoride utilization by dentists, students, and health personnel. After its application, the findings revealed that almost half of the respondents did not have proper information or knowledge about fluoride sources or the daily consumption of fluoride water and dentifrices.
