# Errata

**DOI:** 10.1590/0103-644020256377-er

**Published:** 2025-09-26

For the Article published as:

Chevitarese, A. B., Leite, K. L. de F., Silva, K. S., Jural, L. A., Rocha, D. R. C. da ., Perazzo, M. F., Cury, J. A., & Maia, L. C.. (2025). Development and validation of a questionnaire about fluoride knowledge. *Brazilian Dental Journal*, *36*, e24-6377. https://doi.org/10.1590/0103-644020256377

Into subtitle **Discussion**, paragraph:

An analysis of our samples revealed that the majority of the respondents were female and from Southeast China, which is a Brazilian region with a high concentration of dentistry schools and a large number of dentists in practice (24).

The term **Southeast China** must be only **Southeast**, changing the text as:

An analysis of our samples revealed that the majority of the respondents were female and from Southeast, which is a Brazilian region with a high concentration of dentistry schools and a large number of dentists in practice (24)

